# Diagnostic accuracy of dual-layer spectral CT for osteolytic vertebral metastases

**DOI:** 10.1007/s00256-025-05023-z

**Published:** 2025-09-06

**Authors:** Simone van der Star, Netanja I. Harlianto, Stéphanie V. de Lange, Jorrit-Jan Verlaan, Arnold M. R. Schilham, Madeleine Kok, Pim A. de Jong, Wouter Foppen

**Affiliations:** 1 Department of Radiology and Nuclear Medicine, University Medical Center Utrecht & Utrecht University, PO Box 85500, 3508 GA Utrecht, the Netherlands; 2 Department of Orthopedic Surgery, University Medical Center Utrecht & Utrecht University, PO Box 85500, 3508 GA Utrecht, the Netherlands; 3 Department of Radiation Oncology, University Medical Center Utrecht & Utrecht University, PO Box 85500, 3508 GA Utrecht, the Netherlands; 4 Department of Radiology and Nuclear Medicine, Rijnstate Arnhem, PO Box 9555, 6800 TA Arnhem, the Netherlands

**Keywords:** Computed tomography, Sensitivity, Specificity, Spine, Metastasis

## Abstract

**Objectives:**

To evaluate whether dual-layer spectral computed tomography, compared with conventional CT, improves diagnostic accuracy for osteolytic vertebral metastases. Furthermore, to investigate the influence of dual-layer CT on the subjective visibility of metastases.

**Materials and Methods:**

In this single-center retrospective study, consecutive patients with an untreated primary tumor who underwent dual-layer CT and either MRI or PET-CT as reference standard within 14 days were included. Two independent observers, blinded to the reference, performed two scorings. First, the conventional CT was scored and the results were recorded. Subsequently, Calcium suppression, monoenergetic (monoE40 and monoE200), and Z-effective reconstructions were added. Subjective visibility was compared to conventional CT using a 5-point Likert scale. Diagnostic accuracy measures were calculated with 95% confidence intervals. Sensitivity and specificity were compared using the McNemar’s test.

**Results:**

Fourteen patients (63 ± 8 years; 64.3% female) and 189 vertebrae were included, with 46 vertebrae showing 58 metastases with a mean diameter of 18 mm (range 5–53 mm). For conventional CT, the sensitivity, specificity, and diagnostic accuracy for observer A and B were, respectively, 57% and 57%, 96% and 90%, 85% and 81%. The diagnostic performance did not improve when using the dual-layer CT reconstructions in addition to conventional CT (*p* ≥ 0.13). MonoE40 improved the subjective visibility of metastases. Interobserver agreement was moderate for conventional CT (ĸ:0.48), and dual-layer CT reconstructions (ĸ:0.41–0.51).

**Conclusion:**

Dual-layer CT reconstructions did not improve diagnostic accuracy for osteolytic vertebral metastases compared with conventional CT, although subjective visibility was improved on low monoenergetic reconstructions.

**Supplementary Information:**

The online version contains supplementary material available at 10.1007/s00256-025-05023-z.

## Introduction

Bone is the third most common site of solid cancer metastases [1, 2]. Depending on the primary tumor, bone metastases can occur as osteolytic, sclerotic, or mixed lesions. The three most prevalent solid cancers with osteolytic metastases are lung, breast (often mixed lytic/sclerotic), and renal cancer [1, 3]. Detection of bone metastases is important for tumor staging, and can guide the aim of therapy. Furthermore, osteolytic vertebral metastases can significantly impair quality of life when not treated timely, as they can lead to pathological fractures, severe pain, and neurological deficits [4].

(Contrast-enhanced) computed tomography (CT) is routinely performed for staging and follow-up of oncological patients due to its widespread availability and relatively low costs. However, two meta-analyses of Liu et al. [5] and Harlianto et al. [6], including lytic and sclerotic metastases, showed that the sensitivity of CT to detect vertebral metastases is limited with 67% and 76%, and significantly lower than that of MRI (91% and 90%), PET-CT (92% and 89%), and SPECT (92% and 92%) for the two studies, respectively. As a consequence, further imaging with PET-CT or MRI in addition to CT is frequently performed depending on the level of suspicion and clinical relevance. Conventional CT (cCT) especially lacks sensitivity to detect asymptomatic vertebral metastases because differences in attenuation between the healthy and affected trabecular bone marrow are small, and because benign inhomogeneity is common.

Dual-energy techniques may improve the visibility and characterization of osteolytic vertebral metastases on CT by utilizing different energy spectra, as each material exhibits a distinct energy-dependent attenuation curve. This concept allows differentiation of materials and generation of several dual-layer spectral CT (DLCT) reconstructions [7]. For example, calcium suppression reconstructions (CaSupp) can suppress the attenuation of bone, while monoenergetic reconstructions (monoE) can increase or decrease the attenuation of specific materials by selecting specific energy levels [8]. It has been suggested that this can aid differentiation between acute and chronic fractures in traumatic spine imaging [9]. For further elaboration on dual-energy principles and techniques, we refer to a review by McCollough et al. [7].

A few previous studies [10–15] investigated the potential added value of DLCT for diagnosing vertebral metastases, but large heterogeneity existed among these studies. For example, different type of metastases (e.g., osteolytic, sclerotic, or mixed) were included, different spectral CT scanners (i.e., dual-layer, fast kV-switching) were used, and the included spectral reconstructions varied. Furthermore, three of these previously mentioned studies did not investigate the visual detection of vertebral metastases, but studied quantitative outcomes measured on predefined lesions for further differentiation [11, 14, 15]. One prior study of Abdullayev et al. [10] did focus on the visual detection of vertebral metastases by investigating CaSupp with different suppression indices using DLCT and a protoype post-processing algorithm. This prior study showed significantly improved diagnostic accuracy for the reconstruction with a low calcium suppression index compared with cCT. However, to the best of our knowledge, no studies have explored the visual detection of osteolytic vertebral metastases using other DLCT reconstructions than CaSupp.

In the current study, we evaluated whether DLCT, compared with cCT, improves diagnostic accuracy for osteolytic vertebral metastases. Furthermore, we aimed to investigate the influence of DLCT on the subjective visibility of metastases.

## Materials and methods

### Ethical statement

All procedures involving human participants in this study were in accordance with the ethical standards of the institutional and/or national research committee and with the 1964 Helsinki declaration and its later amendments or comparable ethical standards. A waiver of written informed consent was obtained for this retrospective study (Vidatum reference number 22U-0073). This diagnostic accuracy study was reported according to the “Standards for the Reporting of Diagnostic accuracy studies” (STARD) [16].

### Study population

**Fig. 1:**
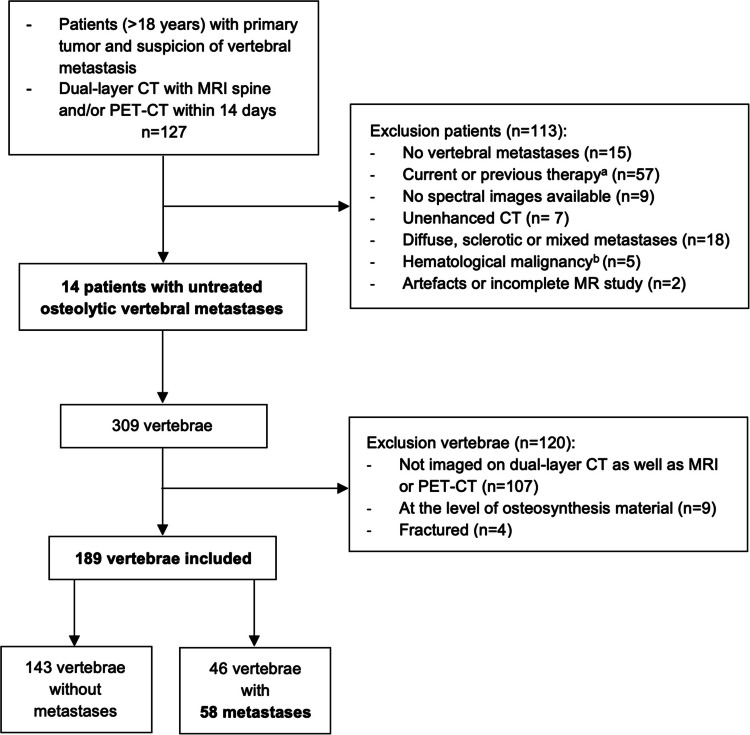
Study flowchart of the included patients with a solid organ malignancy, and the number of vertebrae studied. *a systemic therapy or local radiotherapy; b: including multiple myeloma and lymphoma*

In this single-center retrospective study, performed at the University Medical Center Utrecht in the Netherlands, patients were identified with a PACS search. All patients (≥ 18 years of age) with an untreated primary tumor who underwent contrast-enhanced DLCT as well as MRI or fluorodeoxyglucose (FDG) PET-CT scan with less than 14 days between them, from May 2017 to May 2024, were included. The inclusion process and exclusion criteria are shown in Fig. 1. A total of 14 patients and 189 vertebrae were included, with 46 vertebrae showing 58 metastases. All consecutive and eligible patients since the introduction of the DLCT scanners in our center were included to obtain the largest possible sample size. We performed a post hoc sample size analysis to assess statistical power. In our previous meta-analysis [6], we reported a per-lesion pooled sensitivity of 76% and specificity of 91% for cCT. A 10% increase in sensitivity using DLCT was considered clinically relevant; therefore, we assumed a expected sensitivity of 86% and maintained a specificity of 91% for the sample size calculation. The disease prevalence in our retrospective dataset was 30% (vertebra-based, as only patients with vertebral metastases were included). We used an α level of 0.05, and no dropout rate was applicable. Using an online sample size calculator based on the formula by Buderer et al., we determined that a sample size of > 155 vertebrae was required [17].

### Image acquisition and reconstruction

All CT scans were performed on DLCT scanners (CT7500 or iQon, Philips Healthcare). Patients were scanned according to the local CT protocols for tumor staging with intravenous contrast (Ultravist, Iopromide 300 mgI/mL; Bayer Healthcare), including the neck, thorax and/or abdomen. Injection protocols were based on body weight and scan duration [18, 19]. Scan timing was performed using bolus tracking with a region of interest (ROI) placed in the aorta. The threshold-delay was 50 s. One out of 14 patients was scanned in arterial phase with a threshold-delay of 8 s. The relevant acquisition parameters are listed in Supplementary Table S1.

The cCT and DLCT images were reconstructed into a slice thickness of 0.9 mm with increments of 0.7 mm. The cCT images were reconstructed by combining data from both detector layers and using iterative reconstruction (iDose^4^, level 3) with a soft kernel (B). Spectral reconstructions were generated using IntelliSpace Portal version 12 (Philips Healthcare).

### Index test

**Fig. 2:**
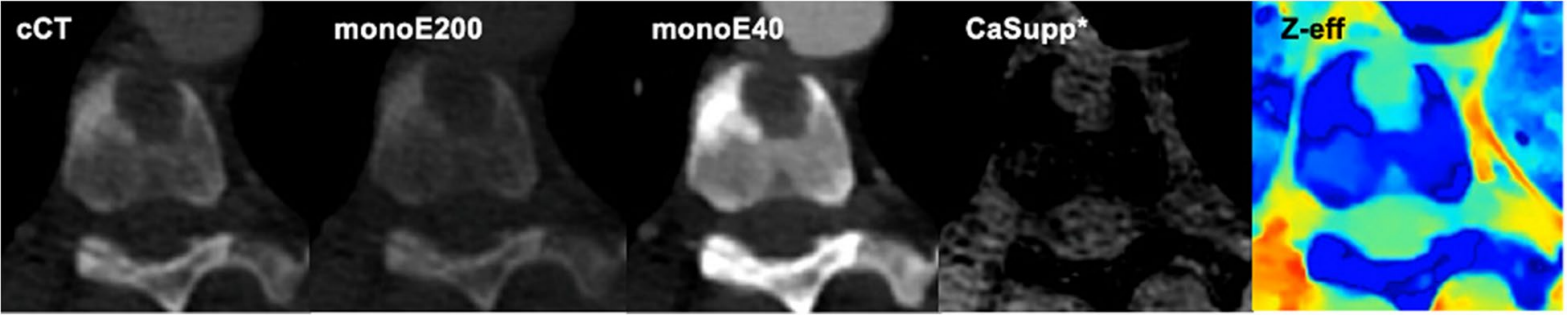
Example of conventional CT and dual-layer CT reconstructions of an osteolytic vertebral metastasis located anteriorly in the vertebral body of Th9 from a 49-year-old female with non-small cell lung cancer. Abbreviations: cCT, conventional CT; monoE200, monoenergetic 200 keV; monoE40, monoenergetic 40 keV; CaSupp, Calcium suppression; Z-eff, Z effective. *calcium suppression index of 55

For our study, both conventional and spectral data were used. The following spectral reconstructions were included (Fig. 2):

- CaSupp. In this reconstruction, calcium-containing voxels are identified using spectral data and replaced by virtual HU-values based on the expected HU-values without calcium. The calcium suppression index depends on the calcium composition weight and can be adjusted manually with a range from 25 to 100. A low index suppresses tissues with low calcium composition weight, and a high index suppresses tissues with high calcium composition weight. The optimal calcium suppression level was left to observer’s discretion and noted in the case record file.
- Z-effective (Z-eff). In this reconstruction, materials are differentiated based on the average atomic number.
- Virtual monoE: 40 keV (monoE40) and 200 keV (monoE200). In contrast to the poly-energetic spectrum from cCT, a specific monoenergetic level from 40 to 200 keV can be selected. In these reconstructions, attenuation of certain materials can be increased. This effect is based on material-specific K-edges, which is an energy level that results in a significant peak in attenuation. Therefore, selecting an energy spectrum around the K-edge of a certain material will result in a significant peak in attenuation of that material compared to the surrounding tissue.

The primary outcome measure was the presence of vertebral metastases on cCT and DLCT reconstructions on a dichotomous scale (yes/no). The size (maximal diameter in sagittal plane) and location within the vertebra (body, pedicle, lamina, spinous process, transverse process) were recorded. In case a metastasis extended over more than one location, the center of the metastasis was registered as the location.

The secondary outcome measure was the subjective visibility of vertebral metastases on DLCT reconstructions compared with cCT using a Likert scale ranging from 1 to 5: 1, only visible on cCT; 2, cCT > DLCT; 3, cCT = DLCT; 4, DLCT > cCT; 5, only visible on DLCT.

The measurements were performed by two board-certified radiologists (WF and SVdL) with 9 and 6 years of CT-experience, respectively. Furthermore, both reader had over five years experience with spectral CT. The readers were blinded to each other’s readings, to the number of metastases in the dataset, and the results of the reference standard. First, the cCT was scored and the results were recorded in the case record file. Subsequently, the DLCT reconstructions were provided to the observers in the same session. The DLCT reconstructions were used as an add-on to cCT and assessed in the following fixed sequence: CaSupp, Zeff, monoE40, monoE200. The observers recorded the following items in the case record file: the localization and size on both cCT and DLCT reconstructions, and the Likert scale of the metastases per DLCT reconstruction compared with cCT.

### Reference standard

MRI and/or FDG PET-CT were used as reference standard for the presence of osteolytic vertebral metastases.

Preferably an MRI was used as reference standard, which was the case in 11 patients. The MRI scans (1.5 T) were performed according to the local vertebral tumor protocol. This protocol consists of the following sequences without intravenous contrast: sagittal and axial T1-weighted turbo spin echo (TSE), 3D T2-weighted TSE, and sagittal T2 short tau inversion recovery (STIR). Metastatic vertebral lesions were defined as: hypointense on T1-weighted images and hyperintense on T2-TSE and STIR images.

When MRI was not available FDG PET-CT was used as a reference standard in 3 patients. FDG PET-CT scans were scanned according to the local tumor protocol for staging. The pre-defined regular fluorodeoxyglucose dose was 20 MBq/kg. Patient preparation consisted of 6 h fasting before the scan. Vertebral lesions on FDG PET-CT were considered positive for metastases in case of focally increased uptake compared with the background uptake of the spine, with or without corresponding lesion on CT, excluding benign etiology such as traumatic fractures or increased uptake of degenerative changes in the endplates and facet joints.

For both MRI and PET-CT, the presence and location of the metastases were based on reports from board-certified radiologists and board-certified nuclear medicine physicians, respectively. In case of multiple metastases without precise description, the presence and location of the metastases were verified by SvdS and a board-certified imager who is both radiologist and nuclear medicine physician (both blinded to the results of the index test).

### Statistical analysis

Baseline characteristics of the patients and vertebral metastases were analyzed using descriptive statistics. Continuous variables were described as mean values and categorical variables as frequencies and percentages.

Analysis for diagnostic performances was vertebra-based. For multiple metastases in one vertebra, each metastasis was included separately. Patient-based analysis was not performed, because only patients with vertebral metastases were included.

For both observers, the true positives, false positives, true negatives, false negatives were recorded. The sensitivity, specificity, positive predictive value (PPV), negative predictive value (NPV), and diagnostic accuracy with 95% confidence intervals (CI) were calculated for cCT and the separate DLCT reconstructions. McNemar’s test was used for comparative analysis of the sensitivity and specificity between cCT and the DLCT reconstructions for each observer. The interobserver agreement was calculated using Cohen’s kappa (ĸ) and the agreement was interpreted as follows: ĸ: 0–0.19 as slight, ĸ: 0.2–0.39 as poor, ĸ: 0.4–0.59 as moderate, ĸ: 0.6–0.79 as good, and ĸ: ≥ 0.8 as excellent.

For the subjective visibility of vertebral metastases on DLCT reconstructions compared with cCT, the median Likert scale was calculated for each DLCT reconstruction. For this analysis, only true positives were included.

The lesion size of the true positives and false negatives were compared using the Mann–Whitney U test. The true positives included the metastases that were detected by one or both observers.

Statistical significance was defined as *p* < *0.05*. Analyses were performed using IBM SPSS statistical software (version 29), and MedCalc software (Diagnostic test evaluation calculator, version 23.0.8) [20].

## Results

###  Baseline characteristics

A total of 14 patients and 189 vertebrae were included, with 46 vertebrae showing 58 metastases. Baseline characteristics of the patients and vertebral metastases are described in Table 1. The mean patient age was 63 years and 64.3% was female. The majority of the primary tumors originated from the breast (n = 3), lung (n = 3), or colon (n = 3). The tumor subtypes are described in Table 1. The mean lesion size was 18 mm (SD: 10 mm), and most metastases were located in the vertebral body (79.3%). The mean time interval between the index test and reference standard was 4 days (range: 0–14 days). 47 (81%) metastases were confirmed by MRI, and 11 (19%) by FDG PET-CT.

**Table 1:** Baseline characteristics of the patients and vertebral metastases

***Patients***	*n* = *14*
Age in years*, mean (SD; range)*	63 (8; 47–78)
Gender*, n (%)*	
Male	5 (35.7)
Female	9 (64.3)
Location primary tumor, *n (histology)*	
Breast	3 (1 ductal, 1 lobular, 1 poorly differentiated)
Lung	3 (2 non-small cell, 1 adeno)
Colon	3 (3 adeno)
Vagina	1 (squamous cell)
Thyroid	1 (follicular)
Oral cavity	1 (squamous cell)
Popliteal region	1 (leiomyosarcoma)
Unknown primary	1 (non-small cell—not otherwise specified)
No. of vertebral metastases per patient*, n*	
1	4
2	2
3	1
4	-
5	3
≥ 6	4
***Osteolytic vertebral metastases***	*n* = *58*
Lesion size in mm*, mean (SD; range)*	18 (10; 5–53)
No. of metastases per vertebrae, *n*	
1	35
2	10
3	1
Location*, n (%)*	
Body	46 (79.3)
Pedicle	8 (13.7)
Lamina	2 (3.4)
Spinous process	1 (1.7)
Transverse process	1 (1.7)
Reference standard, *n (%)*	
MRI	47 (81.0)
PET-CT	11 (19.0)
Time interval between the index test and reference standard in days,* mean (range)*	4 (0–14)

### Diagnostic accuracy of conventional CT and spectral CT reconstructions to detect osteolytic vertebral metastases

The true positives, false positives, true negatives, and false negatives are listed in Supplementary Table S2. On cCT, the sensitivity, specificity, and diagnostic accuracy for observer A and B were 57% and 57% (CI:43–70), 96% (CI:92–99) and 90% (CI:84–94), 85% (CI:80–90) and 81% (CI:75–86), respectively (Table 2). The PPV and NPV for observer A was 85% (CI:71–93) and 85% (CI:81–89). The PPV and NPV for observer B was 69% (CI:56–79) and 85% (CI:80–88). For both observers, the sensitivity, specificity, PPV, NPV, and diagnostic accuracy did not improve when using DLCT reconstructions in addition to cCT (Table 2). Exclusion of the arterial phase scan did not show new insights (Supplementary Table S3 and S4).

**Table 2:** Sensitivity, specificity, positive predictive value, negative predictive value, and diagnostic accuracy with 95% confidence intervals for conventional CT and dual-layer CT reconstructions for both observers for the detection of osteolytic vertebral metastases

	Sensitivity	*p*-value^1^	Specificity	*p*-value^a^	PPV	NPV	Accuracy
**Observer A**							
CCT	57 (43–70)		96 (92–99)		85 (71–93)	85 (81—89)	85 (80–90)
CaSupp^b^	40 (27–53)	0.002*	97 (92–99)	1.00	82 (65–92)	81 (77—84)	81 (75–86)
MonoE40	57 (43–70)	1.00	95 (91–98)	1.00	83 (69–91)	85 (81–89)	85 (79–89)
MonoE200	57 (43–70)	1.00	96 (92–99)	1.00	85 (71–93)	85 (81–89)	85 (80–90)
Z-effective	53 (40–67)	0.50	95 (91–98)	1.00	82 (67–90)	84 (80–88)	84 (78–88)
**Observer B**							
CCT	57 (43–70)		90 (84–94)		69 (56–79)	85 (80–88)	81 (75–86)
CaSupp^b^	53 (40–67)	0.69	85 (78–90)	0.12	57 (46–68)	83 (79–87)	77 (70–82)
MonoE40	64 (50–76)	0.13	85 (78–90)	0.008*	62 (51–71)	86 (82–90)	79 (73–85)
MonoE200	59 (45–71)	1.00	92 (87–96)	0.45	74 (61–84)	86 (81–89)	83 (78–88)
Z-effective^c^	-	-	-	-	-	-	-

Diagnostic values are percentages with 95% confidence intervals in parentheses

PPV: positive predictive value; NPV: negative predictive value; CCT: Conventional CT; CaSupp: Calcium Suppression

a: Comparative analysis for sensitivity and specificity between conventional CT alone, and dual-layer CT reconstruction in addition to conventional CT using McNemar’s test. Asterisks indicate statistically significant differences

b: The mean preferred calcium suppression index was 55 (SD: 5; range: 50–75) for observer A, and 57 (SD: 20; range: 25–100) for observer B

c: Scored as non-diagnostic in 13 out of 14 patients

Overall, observer B scored more true positives, but also more false positives on DLCT reconstructions in addition to cCT compared with observer A (Supplementary Table S2).

For monoE40, the specificity of observer B was significantly lower compared with cCT (85% (CI:78–90) vs. 90% (CI:84–94), *p* = 0.008). In contrary, the sensitivity of observer B for monoE40 was higher compared with cCT (64% (CI:50–76) vs. 57% (CI:43–70)), although not significantly different (*p* = 0.13).

For Z-eff, observer B labeled this reconstruction as non-diagnostic in 13 out of 14 patients, whereas observer A did not show statistically significant differences compared with cCT.

The interobserver agreement was moderate for cCT (ĸ = 0.48) as well as for the DLCT reconstructions (CaSupp and monoE40, ĸ = 0.41; monoE200, ĸ = 0.51) (Table 3).

**Table 3:** The interobserver agreement for conventional CT and dual-layer spectral CT reconstructions

	Cohen’s ĸ (95% CI)
Conventional CT	0.48 (0.33–0.63)
Calcium suppression	0.41 (0.27–0.55)
MonoE40	0.41 (0.27–0.55)
MonoE200	0.51 (0.37–0.65)
Z-effective*	-

*CI* confidence-interval

^*^ Observer B scored this reconstruction as non-diagnostic in 13 out of 14 patients

When comparing the lesion size of the true positives for one or both observers (n = 42) with the false negatives (n = 16), the lesion size of the true positives was significantly larger with 20 mm (SD: 10; range: 6–53) versus 10 mm (SD: 5, range: 5–21) (p < 0.001). Regarding the location, most metastases were in the vertebral body, for both the true positives (81%) and the false negatives (75%).

### Subjective visibility of vertebral metastases on spectral CT reconstructions

**Fig. 3:**
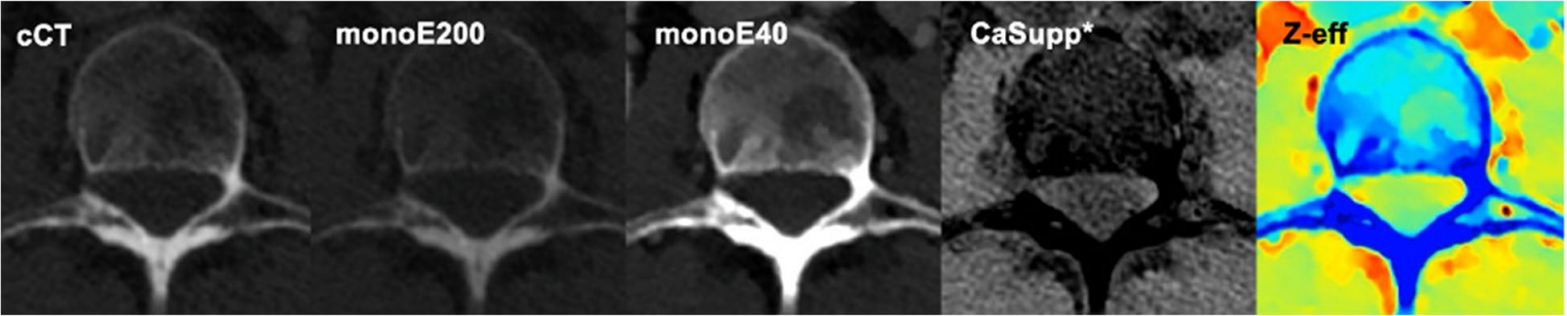
Conventional CT and dual-layer CT reconstructions of a 74-year-old female with breast cancer showing a vertebral osteolytic metastasis left posteriorly in the vertebral body of L3. It was best visible on monoE40, showed comparable visibility on Z-effective, reduced visibility on monoE200, and was not delineated on the Calcium suppression reconstruction. Abbreviations: cCT, conventional CT; monoE200, monoenergetic 200 keV; monoE40, monoenergetic 40 keV; CaSupp, Calcium suppression; Z-eff, Z-effective. *Calcium suppression index of 55

The subjective visibility was compared to cCT and scored on a Likert scale ranging from 1 to 5: 1, only visible on cCT; 2, cCT > DLCT; 3, cCT = DLCT; 4, DLCT > cCT; 5, only visible on DLCT. MonoE40 showed improved visibility of vertebral metastases compared with cCT with a median score of 4 for both observers on monoE40 (Table 4). The other spectral reconstructions did not improve the subjective visibility of vertebral metastases. For CaSupp and monoE200, observer A labeled the visibility of metastases as inferior to cCT (Likert score 2) and observer B categorized the visibility as equal to cCT (Likert score 3). On Z-eff, the visibility of metastases did not improve compared with cCT according to observer A (Likert score 3). Figure 3 shows an example image of a vertebral metastasis that was best visible on monoE40, showed comparable visibility on Z-eff, reduced visibility on monoE200, and was not delineated on CaSupp.

**Table 4:** Subjective visibility of vertebral metastases on dual-layer spectral CT reconstructions compared with conventional CT using a Likert scale

	TP *n*	Only on cCT (1)	cCT > DLCT (2)	cCT = DLCT (3)	DLCT > cCT (4)	Only on DLCT (5)	Median Likert (1–5)
**Observer A**							
CaSupp, *n (%)*	33	10 (30.3)	15 (45.5)	7 (21.2)	1 (3.0)	0 (0)	2
MonoE40, *n (%)*	33	0 (0)	0 (0)	15 (45.5)	18 (54.5)	0 (0)	4
MonoE200, *n (%)*	33	0 (0.0)	33 (100.0)	0 (0)	0 (0)	0 (0)	2
Z-effective, *n (%)*	33	2 (6.1)	13 (39.4)	15 (45.5)	3 (9.1)	0 (0)	3
**Observer B**							
CaSupp *n (%)*	35	4 (11.4)	12 (34.3)	14 (40.0)	3 (8.6)	2 (5.7)	3
MonoE40, *n (%)*	37	0 (0)	0 (0)	14 (37.8)	19 (51.4)	4 (10.8)	4
MonoE200, *n (%)*	35	1 (2.9)	6 (17.1)	25 (71.4)	1 (2.9)	2 (5.7)	3
Z-effective^a^, *n (%)*	1	-	-	1	-	-	

*TP* true positives, *cCT* conventional CT; *DLCT* dual-layer CT; *CaSupp* Calcium suppression

a: Scored as non-diagnostic in 13 out of 14 patients

The sum of the percentages in the rows is 100%

**Fig. 4:**
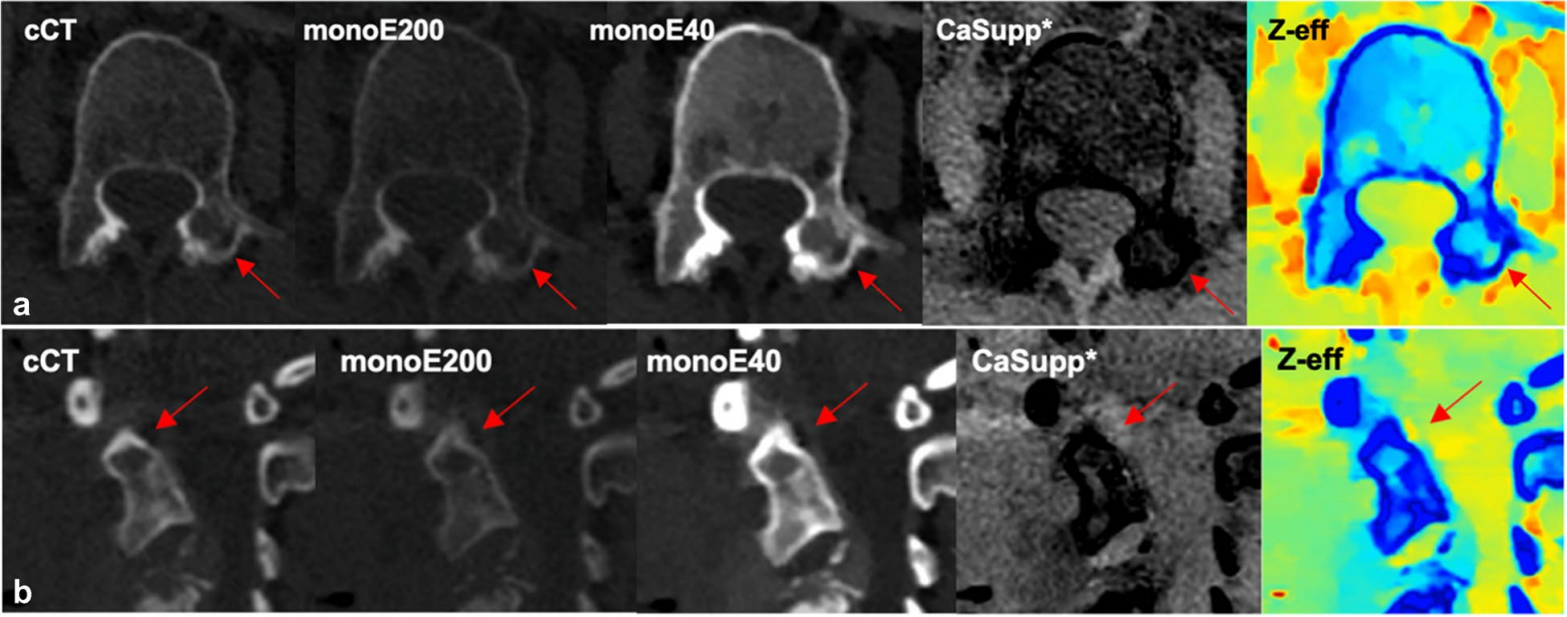
Conventional CT and dual-layer CT reconstructions of two metastases from different patients in the left pedicle of L2 and the odontoid process of C2 that were only detected by observer B on monoE40 and monoE200 (**a**), and monoE40 (**b**), respectively. Abbreviations: cCT, conventional CT; monoE200, monoenergetic 200 keV; monoE40, monoenergetic 40 keV; CaSupp, Calcium suppression; Z-eff, Z-effective. *Calcium suppression index of 55

Only observer B scored four vertebral metastases as’only visible on DLCT’ (Likert score 5), mainly on monoE40. Two out of four metastases were also detected by observer A on both cCT and DLCT reconstruction. The other two metastases, located in the pedicle of a L2 vertebra (14 mm) and in an odontoid process of C2 (8 mm), were only detected on the monoE40 and monoE200, and monoE40, respectively (Fig. 4).

## Discussion

Our results showed that dual-layer spectral CT reconstructions (CaSupp, Z-eff, monoE40, monoE200) in addition to conventional CT reconstructions did not significantly improve the diagnostic accuracy for osteolytic vertebral metastases. Some benefit of monoE40 cannot be excluded as monoE40 did show improved subjective visibility compared with conventional CT. However, the monoE40 showed a significantly lower specificity compared with conventional CT for one observer.

To the best of our knowledge, this is the first study investigating monoE reconstructions for the detection of osteolytic vertebral metastases. Prior studies [21–23] mainly focused on the use of monoE reconstructions for metal artefact reduction in the spine. Our study demonstrated improved subjective visibility of metastases on monoE40. This can be explained by the low K-edge of 4 keV of calcium, causing increased attenuation of the bone and greater contrast between the metastases and surrounding bone. The improved visibility did not result in substantially improved diagnostic performances. However, the improved subjective visibility may be useful in clinical practice for easier identification of osteolytic vertebral metastases. For this reason, our department implemented low monoE reconstructions alongside cCT reconstructions in the routine workflow for oncological scans performed for staging or follow-up purposes, which may also benefit organs other than bone.

In contrast to monoE40, high monoE reconstructions will reduce the attenuation of bone. Therefore, we hypothesized that on monoE200 the combination of suppressed bone attenuation and edema-like changes of the metastases may improve the contrast between the bone and metastases. Our findings did not confirm this hypothesis as monoE200 did not demonstrate improved visibility or diagnostic accuracy. This may be explained by the iodine content of enhancing metastases. The K-edge of iodine is low (33 keV) which also results in lower attenuation of enhancing metastases on high monoE reconstructions.

For CaSupp, we found no positive effect on the diagnostic accuracy or subjective visibility. Moreover, one of the observers reported reduced subjective visibility of the metastases. One prior study of Abdullayev et al. [10] also examined the use of CaSupp for the detection of vertebral metastases. In contrast to our study, where the calcium suppression index was left to observer’s discretion, they examined two separate CaSupp reconstructions with a fixed low (25) and medium (50) index for the subjective analysis using prototype software. In our study, the mean chosen index of the observers was 55 and 57, probably comparable to the medium index (50). For the medium index, they reported a reduced sensitivity from 78 to 71%, and a slightly increased specificity from 85 to 88%. The small differences reflect our findings with a decreased sensitivity from 57 to 40% for one of the observers, without a significant increase in specificity. For the low index, they reported improved diagnostic performances with a significantly increased sensitivity and specificity from 78 to 85%, and from 82 to 84%, respectively. We could not confirm these results. Although the observers in our study were free to choose the optimal suppression index, they predominantly selected indices within the medium range. Some heterogeneity in CaSupp reconstructions between both studies may exist, because Abdullayev et al. used a prototype post-processing algorithm. Overall, the accuracy was higher in their study. The differences in the diagnostic performances between the studies may be caused by differences in the prevalence of vertebral metastases. In the study of Abdullayev et al., 60.4% of the included vertebrae (203/336) were metastatic with an unknown number of metastases, and in our study 24.3% of the included vertebrae (46/189) were metastatic with 58 metastases. Furthermore, their analysis was purely vertebra-based as they did not consider the number of detected metastases per vertebra.

In our study, both observers demonstrated differences in the interpretation of the Z-eff reconstruction. One observer reported similar diagnostic performances and subjective visibility compared with cCT, while the other observer classified the Z-eff as non-diagnostic. This is caused by a different interpretation of the diagnostic value. When using Z-eff in addition to cCT, the metastases are recognizable. However, if Z-eff would be interpretated separately, the metastases cannot be distinguished from other parts of healthy bone marrow showing similar appearances. No previous literature on Z-eff exists for this diagnostic question. Furthermore, the Z-eff reconstruction is not routinely used in clinical practice, leading to less experience among the readers compared to other spectral reconstructions. This may have contributed to the discrepancy in subjective visibility between the two readers for the Z-eff reconstruction.

Although it is known that metastases show enhancement due to tumor angiogenesis [24, 25], we did not include iodine-only reconstructions. Borggrefe et al. [11] examined the iodine concentration on iodine-only reconstructions as a quantitative biomarker for the differentiation of healthy bone marrow and metastases. They demonstrated significant differences in iodine concentrations, however, with substantial overlap. This reflects our clinical experience as both red bone marrow and osteolytic metastases show enhancement, making the visual differentiation between healthy bone marrow and metastases not possible in our hands.

A prior study from Buus et al. [26] evaluated the diagnostic accuracy of DLCT compared to MRI and cCT in metastatic breast cancer, including bone lesions from the chest to pelvis. Available spectral reconstructions included MonoE (40–200 keV), Z-effective, iodine-only, and virtual noncontrast. MRI showed the highest diagnostic accuracy (sensitivity of 86%, specificity of 98%). Consistent with our findings, DLCT (sensitivity 77%, specificity 93%) showed no statictically significant difference compared to cCT (sensitivity 74%, specificity 95%).

Our study showed a lower sensitivity of cCT (57%) compared to two meta-analyses of Liu et al. (67%) and Harlianto et al. (76%) [5, 6]. This may be caused by the retrospective inclusion of patients that underwent a CT scan, and MRI or FDG PET/CT-scan within 14 days of each other, introducing a selection bias of patients with indeterminate more challenging lesions needing further imaging. Moreover, we did not exclude metastases with a small lesion size (< 10 mm). The lesion size of the false negatives was significantly smaller (10 mm, range 5–21 mm) compared with the true positives (20 mm; range: 6–53 mm).

We solely included osteolytic vertebral metastases, but we also included three patients with breast cancer while these tumors typically show mixed metastases. In our study, most breast cancer cases were excluded due to the presence of mixed lytic/sclerotic or sclerotic metastases.

A strength of our study is that we focused on detecting challenging osteolytic metastases with a decent reference standard and with experienced readers. Also, our scanning and reading protocol reflects clinical practice. The majority of the included CT scans (13 out of 14) were scanned in the portal venous phase, similar to scans for oncological staging purposes. Furthermore, the DLCT reconstructions were provided simultaneously with cCT, and the observers were allowed to adjust the window level and window width.

Some limitations of our study should also be mentioned. Firstly, we used MRI and FDG PET-CT scans as reference standard, because it was not possible to obtain histological confirmation for each vertebral metastasis. Secondly, the observers were exclusively focused on detecting vertebral metastases which introduces a review bias. However, this did not result in increased sensitivity compared with prior studies. Also, low monoE reconstructions may have stronger benefits in less experienced readers, due to improved subjective visibility on low monoE compared to cCT. Thirdly, the DLCT reconstructions were scored in the same session, which could have influenced the interpretation due to knowledge of the other reconstructions. Nevertheless, this way of reading is also in line with current practice. Another limitation is that we performed the CT scans on a DLCT. This limits the generalizability of our findings to other types of spectral CT scanners. Finally, our sample size was small, comprising only 14 patients. However, we conducted a vertebra- and lesion-based analysis of 189 vertebrae with 58 metastases, which provided sufficient statistical power for our analysis. Future studies with larger patient cohorts and prospective designs are warranted to further investigate the diagnostic accuracy of spectral CT for detecting osteolytic vertebral metastases, as we did observe some effect of MonoE40.

In conclusion, dual-layer spectral CT reconstructions did not improve diagnostic accuracy for osteolytic vertebral metastases compared with conventional CT, although some benefit of monoE40 cannot be excluded due to improved subjective visibility.

## Supplementary Information

Below is the link to the electronic supplementary material.

Supplementary file1 (DOCX 21 KB)

## Data Availability

The datasets used and analysed during the current study are available from the corresponding author on reasonable request.

## Abbreviations

cCT: Conventional CT
DLCT: Dual-layer spectral CT
CaSupp: Calcium suppression
monoE40: Monoenenergetic 40 keV
monoE200: Monoenenergetic 200 keV
Z-eff: Z-effective
TSE: Turbo spin echo
STIR: Short tau inversion recovery
