# Supplementary material for: Diagnostic accuracy of dual-layer spectral CT for osteolytic vertebral metastases
Source: Skeletal Radiol. 2025 Sep 6;55(2):329–39. doi: 10.1007/s00256-025-05023-z (PMC12743067; doi:10.1007/s00256-025-05023-z)
Supplement: Supplementary file 1 — Supplementary file1 (DOCX 21 KB) [file 256_2025_5023_MOESM1_ESM.docx]

**Supplementary materials**

**Supplementary table S1:** Acquisition parameters of the dual-layer spectral CT scanners^a^

|  | iQon (n=7) | CT7500 (n=7) |
| --- | --- | --- |
| Collimation (mm)  Matrix  Tube voltage (kV)  Tube current (mAs)  Pitch  Gantry rotation time (s)  Reconstruction kernel  Slice thickness/increment (mm) | 64 x 0.625 mm  512x512  120  Ref: 73 - 104  1.258  0.27  B (iDose^4^)  0.9/0.7 | 128 x 0.625 mm  512x512  120  Ref: 70  1.15 - 1.39  0.27 - 0.33  B (iDose^4^)  0.9/0.7 |

a: CT7500 or iQon spectral CT (Philips Healthcare, Best, The Netherlands)

**Supplementary Table S2**: Diagnostic results of conventional CT and dual-layer spectral reconstructions for detecting osteolytic vertebral metastases.

|  | True positives  *n* | False negatives  *n* | True negatives  *n* | False positives  *n* |
| --- | --- | --- | --- | --- |
| **Conventional CT**  Observer A  Observer B  **Calcium suppression**  Observer A  Observer B  **MonoE40**  Observer A  Observer B  **MonoE200**  Observer A  Observer B  **Z-effective**  Observer A  Observer B^a^ | 33  33  23  31  33  37  33  34  31  1 | 25  25  35  27  25  21  25  24  27  - | 145  139  146  131  144  131  145  142  144  6 | 6  15  5  23  7  23  6  12  7  5 |

a: Scored as non-diagnostic in 13 out of 14 patients.

**Supplementary Table S3**: Diagnostic results of conventional CT and dual-layer spectral CT reconstructions for detecting osteolytic vertebral metastases with exclusion of the arterial phase scan.

|  | True positives  *n* | False negatives  *n* | True negatives  *n* | False positives  *n* |
| --- | --- | --- | --- | --- |
| **Conventional CT**  Observer A  Observer B  **Calcium Suppression**  Observer A  Observer B  **MonoE40**  Observer A  Observer B  **MonoE200**  Observer A  Observer B  **Z-effective**  Observer A  Observer B^a^ | 29  29  21  29  29  33  29  30  27  1 | 18  18  26  18  18  14  18  17  20  - | 145  139  146  131  144  131  145  142  144  6 | 6  15  5  23  7  23  6  12  7  5 |

a: Scored as non-diagnostic in 13 out of 14 patients.

**Supplementary Table S4:** Sensitivity, specificity, positive predictive value, negative predictive value, and diagnostic accuracy with 95% confidence intervals for conventional CT and spectral CT reconstructions for both observers for the detection of osteolytic vertebral metastases with exclusion of the arterial phase scan.

|  | Sensitivity | p-value^1^ | Specificity | p-value^a^ | PPV | NPV | Accuracy |
| --- | --- | --- | --- | --- | --- | --- | --- |
| **Observer A**  CCT  CaSupp^b^  MonoE40  MonoE200  Z-effective | 62 (46 – 75)  45 (30 – 60)  62 (46 – 76)  62 (46 – 75)  57 (42 – 72) | 0.008*  1.00  1.00  0.50 | 96 (92 – 99)  97 (92 – 99)  95 (91 – 98)  96 (92 – 99)  95 (91 – 98) | 1.00  1.00  1.00  1.00 | 83 (68 – 92)  81 (63 – 91)  81 (66 – 90)  83 (68 – 92)  79 (62 – 89) | 89 (85 – 92)  85 (81 – 88)  89 (85 – 92)  89 (85 – 92)  88 (84 – 91) | 88 (83 – 92)  84 (79 – 89)  87 (82 – 92)  88 (83 – 92)  86 (81 – 91) |
| **Observer B**  CCT  CaSupp^b^  MonoE40  MonoE200  Z-effective^c^ | 62 (46 – 75)  62 (46 – 75)  70 (55 – 83)  64 (49 – 77)  - | 1.00  0.13  1.00  - | 90 (84 – 94)  85 (78 – 90)  85 (78 – 90)  92 (87 – 96)  - | 0.12  0.008*  0.45  - | 66 (53 – 77)  56 (45 – 66)  59 (49 – 69)  71 (58 – 82)  - | 88 (84 – 92)  88 (83 – 91)  90 (86 – 94)  89 (85 – 92)  - | 84 (78 – 88)  80 (73 – 85)  82 (76 – 87)  86 (80 – 90)  - |

Diagnostic values are percentages with 95% confidence intervals in parentheses

PPV: positive predictive value; NPV: negative predictive value; CCT: Conventional CT; CaSupp: Calcium Suppression

a: Comparative analysis for sensitivity and specificity between conventional CT alone, and spectral CT reconstruction in addition to conventional CT using McNemar’s test. Asterisks indicate statistically significant differences.

b: The mean preferred calcium suppression index was 55 (SD: 5; range: 50-75) for observer A, and 57 (SD: 20; range: 25-100) for observer B.

c: Scored as non-diagnostic in 13 out of 14 patients.
